# HLA-E Presents Glycopeptides from the *Mycobacterium tuberculosis* Protein MPT32 to Human CD8^+^ T cells

**DOI:** 10.1038/s41598-017-04894-0

**Published:** 2017-07-04

**Authors:** Melanie J. Harriff, Lisa M. Wolfe, Gwendolyn Swarbrick, Megan Null, Meghan E. Cansler, Elizabeth T. Canfield, Todd Vogt, Katelynne Gardner Toren, Wei Li, Mary Jackson, Deborah A. Lewinsohn, Karen M. Dobos, David M. Lewinsohn

**Affiliations:** 1 Veterans Administration Portland Health Care System, Research & Development, 3710 SW US Veterans Hospital Road, Portland, OR 97239 USA; 2 Oregon Health & Sciences University, Department of Pulmonary and Critical Care Medicine, 3181 SW Sam Jackson Park Road, Portland, OR 97239 USA; 3 Colorado State University, Mycobacteria Research Laboratories, Department of Microbiology, Immunology, and Pathology, 1682 Campus Delivery, Fort Collins, CO 80523 USA; 4 Oregon Health & Sciences University, Department of Pediatrics, 3181 SW Sam Jackson Park Road, Portland, OR 97239 USA

## Abstract

Infection with *Mycobacterium tuberculosis* (Mtb), the bacterium that causes tuberculosis, remains a global health concern. Both classically and non-classically restricted cytotoxic CD8^+^ T cells are important to the control of Mtb infection. We and others have demonstrated that the non-classical MHC I molecule HLA-E can present pathogen-derived peptides to CD8^+^ T cells. In this manuscript, we identified the antigen recognized by an HLA-E-restricted CD8^+^ T cell clone isolated from an Mtb latently infected individual as a peptide from the Mtb protein, MPT32. Recognition by the CD8^+^ T cell clone required N-terminal O-linked mannosylation of MPT32 by a mannosyltransferase encoded by the Rv1002c gene. This is the first description of a post-translationally modified Mtb-derived protein antigen presented in the context of an HLA-E specific CD8^+^ T cell immune response. The identification of an immune response that targets a unique mycobacterial modification is novel and may have practical impact in the development of vaccines and diagnostics.

## Introduction

Tuberculosis (TB), caused by infection with *Mycobacterium tuberculosis* (Mtb), remains a leading cause of infectious disease morbidity and mortality worldwide, with 10.4 million new cases, including nearly 500,000 multidrug-resistant (MDR-TB) cases, and 1.8 million deaths in 2015 according to the 2016 Global Tuberculosis Report (World Health Organization). The lack of an effective vaccine, the emergence of MDR-TB, and co-infection with HIV have hampered the effort to eradicate TB. Nonetheless, most people infected with Mtb successfully contain the infection. Critical to this containment is a robust, TH1-type cellular immune response. While CD4^+^ T cells are essential in containing Mtb infection, CD8^+^ T cells play a unique role through their ability to preferentially recognize and eliminate heavily infected cells^1^, as well as their ability to recognize and lyse Class II negative cells^2, 3^. Furthermore, CD8^+^ T cells are particularly important in mouse^4^ and non-human primate^5^ models of persistent Mtb infection, highlighting the importance of eliciting a CD8^+^ T cell response during vaccination.

CD8^+^ T cell antigens are presented on classical (MHC Class Ia) or non-classical (MHC Class Ib) molecules. Classical Class I molecules, encoded by HLA-A, -B, and –C^6^, are highly polymorphic and present a highly diverse array of specific peptide sequences. Non-classical Class I molecules, on the other hand, have limited polymorphism and present pathogen modified antigens or altered self-ligands in the context of infection. For example, CD1 molecules present glycolipids^7^ and MR1 molecules present Vitamin B metabolites^2, 8^. Additionally, while CD8^+^ T cells restricted by classical Class I molecules undergo clonal expansion and require time to manifest their effector responses, CD8^+^ T cells restricted by non-classical Class I molecules such as MR1 are immediately able to elicit effector mechanisms upon encounter with antigen. The human CD8^+^ T cell response to Mtb infection has been shown to involve antigen presented on both classical and non-classical Class I molecules. HLA-E is a non-classical Class I molecule that was originally described as binding ligands for NK receptors^9–11^. However, many have shown that HLA-E also restricts αβ T cells that respond to pathogen-derived antigens^12–15^.

HLA-E has been well-studied in the context of its ability to regulate NK cell activation through interaction with CD94-NKG2 receptors. In this regard, HLA-E binds leader sequence peptides derived from Classical MHC-I molecules, and the levels of HLA-E cycling to and from the surface are regulated by the availability of MHC-I leader sequences^9^. When MHC-I expression is disrupted, such as during a viral infection, the altered HLA-E surface expression is recognized by NK cells via the CD94-NKG2 receptor, and the cell is more susceptible to NK cell lysis. Surprisingly little is known about the pathogen-derived ligands for HLA-E, and how these ligands contribute to interaction with the T cell receptor (TCR) of HLA-E restricted αβ CD8^+^ T cells. A limited number of studies have shown that there is recognition of pathogen-derived ligands presented on HLA-E that occurs through interaction with the TCR^12–14, 16^. For example, HLA-E restricted T cells recognize peptides from the GroEL protein of *Salmonella typhi* and secrete granzyme B and IFNγ in response to *S. typhi*-infected target cells^16^. Joosten *et al*.^15^ used bioinformatics analysis and subsequent functional characterization to identify potential HLA-E ligands from Mtb, and we have also recently identified HLA-E ligands presented in the context of Mtb infection (unpublished data). Both studies identified numerous peptides that were recognized by CD8^+^ T cells from PPD+ donors^15^, as well as TB patients and healthy donors (unpublished data). Interestingly, recent studies analyzing T cell responses to predicted Mtb HLA-E epitopes demonstrate that HLA-E restricted CD8^+^ T cells can have a Th2 phenotype^17, 18^. These studies underscore the potential importance of HLA-E in the response to Mtb.

The limited polymorphism of HLA-E and lack of down-regulation during HIV infection makes the continued identification of Mtb-specific HLA-E antigens an attractive goal in developing novel vaccine candidates or diagnostic targets. In fact, recent studies demonstrate that HLA-E plays an important role in the expansion of SIV-reactive T cells following vaccination of rhesus macaques^19^. Therefore, we used proteomics and mass spectrometry coupled with immunological assays to identify the cognate antigen for a previously described HLA-E restricted CD8^+^ T cell clone isolated from a latently infected individual (D160 1–23)^13^. We show that the antigen associated with the D160 1–23 T cell clone is the post-translationally modified glycoprotein MPT32 (also called Rv1860 or Apa), and that the epitope for the clone relies on N-terminal O-linked mannosylation of this protein. This is the first description of a glycopeptide ligand associated with HLA-E, and has implications with regard to identification of additional pathogen-relevant HLA-E ligands.

## Results

### Proteomic characterization of Mtb subcellular fractions recognized by a human HLA-E restricted T cell clone, D160 1-23

**Figure 1:**
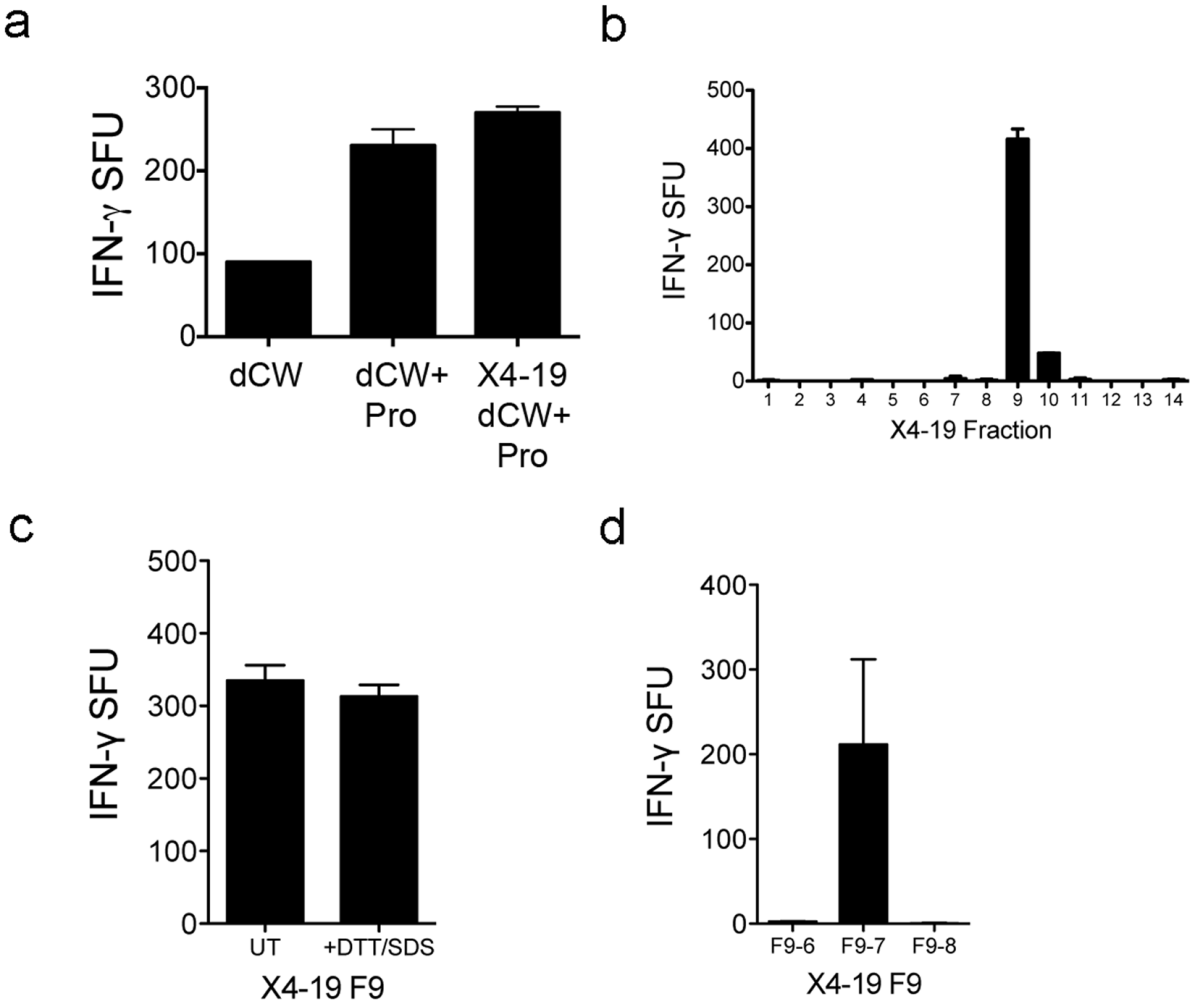
D160 1–23 T cell activity is contained in RP-HPLC fractions that contain the Mtb protein MPT32 (Rv1860). (**a**–**d**) Human monocyte-derived dendritic cells (DC) were pulsed with the following samples and used as antigen presenting cells in an ELISPOT assay with IFN-γ release (spot forming units (SFU)) by the HLA-E restricted T cell clone, D160 1–23, as a readout: (**a**) Delipidated cell wall (dCW) or pronase-digested dCW (dCW + Pro) from H37Rv Mtb, or pronase-digested dCW from the X4–19 ΔHspX Mtb mutant. (**b**) RP-HPLC fractions from the X4-19 ΔHspX Mtb mutant. (**c**) SDS and DTT-treated X4-19 Fraction 9 (F9). (**d**) Extended RP-HPLC fractions of X4-19 F9.

We previously isolated an HLA-E restricted CD8^+^ T cell clone from a latently infected individual (D160 1–23)^13^. To identify potential HLA-E specific antigens, subcellular fractions generated from Mtb whole cell lysate and the culture filtrate were tested against D160 1–23 in IFN-γ ELISPOT assays. The activity of this T cell clone was unique to the cell wall fraction and was enhanced when tested against cell wall material subjected to organic extraction to remove non-covalently cell wall associated lipids (delipidated cell wall, dCW) (Fig. 1a). Proteolytic digestion of dCW by the non-specific protease pronase resulted in further enhancement of T cell activation (Fig. 1a). Digestion of dCW with trypsin abrogated the response of D160 1–23 (data not shown). Reverse phase HPLC fractionation of pronase-digested dCW successfully isolated biological activity to a single peptide fraction. Preliminary LC-MS/MS analysis of the peptides isolated to this fraction identified several proteins, with four related peptides confidently identified from the protein chaperone HspX (Table 1).

**Table 1:** Peptides identified from LC-MS analysis of pronase-digested dCW active fraction.

Protein accession number	Protein name	Protein identification probability*	Peptide sequence	Peptide identification probability*
Rv0578c	PE-PGRS	99.6%	GGAGGAGGNGIAGVTGTSAST	95.0%
			GWLYGNGGAGGFGGAGAVGGNGGAGGTAGLFGVGGAG	91.5%
Rv2031c	hspX	99.9%	AELPGVDPDKDVDI	95.0%
			ELPGVDPDKDVDI	95.0%
Rv2816c	Hypothetical protein Rv2817c	96.8%	DVAESIRTMKHSLAWVDRSG	95.0%
Rv3512	PE_PGRS	96.9%	ITGGTAGTAGAAGNGGAAGKGGAGGQGGTGG	95.0%
Rv3825c	Pks2	98.9%	TVGMVEAHGPGTPIGDPIEYASVSEVYGV	95.0%

^*^Proteins and peptides were accepted as potential candidates if probability scores were calculated as greater than or equal to 95% and 90%, respectively.

### T cell reactivity is contained to RP-HPLC fractions that contain the Mtb protein MPT32 (Rv1860)

Synthetic analogs of overlapping peptides from HspX were tested for their ability to activate the D160 1–23 clone; however, no T cell activity was identified for these synthetic reagents (data not shown). Interrogation of native HspX demonstrated that neither this protein, nor any of its peptides, elicited a response by D160 1–23 (data not shown). As we and others have demonstrated that biological activity is associated with the native protein as a result of its chaperone function^20^, an HspX knockout mutant, X4-19 ΔHspX, was used to determine whether HspX chaperoned the cognate antigen or was essential to retain/stabilize the biologically active product. From these studies we confirmed that T cell reactivity was enhanced in pronase-digested delipidated cell wall from the X4-19 ΔHspX strain of Mtb (Fig. 1a). This validated that the HspX protein does not contain the HLA-E ligand for D160 1–23 and was not required for its activity; thus the X4-19 ΔHspX strain was used for all subsequent experiments to remove this confounding protein.

RP-HPLC fractionation and testing for T cell activation was repeated with pronase-digested whole cell lysate (WCL) from the X4-19 ΔHspX strain, and biological activity was consistently reduced primarily to one fraction, F9 (Fig. 1b). LC-MS analyses of active fractions were used to generate a consensus peptide list (Table 2). Again, none of these candidate proteins, produced as synthetic peptide reagents, nor recombinant or native proteins, demonstrated biologic activity when tested (data not shown). To further resolve the source of the observed T cell activity, the active fraction was subjected to additional treatments. Boiling with 4% SDS and 1 mM DTT for 30 min to reduce the proteins did not ablate IFNγ secretion by D160 1–23 (Fig. 1c). Additional RP-HPLC of F9 on an extended gradient of acetonitrile associated T cell activity to sub-fraction F9-7 (Fig. 1d). Peptide fragments ranging from 700 Da to 2100 Da were visualized in Fraction F9-7 using matrix-assisted laser desorption ionization, time-of-flight (MALDI-TOF) mass spectrometry. Through complementary analyses of sub-fraction F9-7 MALDI-TOF and F9 LC-MS data, peptide sequences could be assigned for several ion species identified by MALDI-TOF (Table 3). This included the identification of several peptides from MPT32 (Rv1860), a protein that was not identified in other fractions.

**Table 2:** Peptides identified from LC-MS analysis of fraction 9 of pronase-digested WCL from the X4-19 ΔHspX Mtb strain.

Protein accession number	Protein name	Observed mass (M + H)	Peptide sequence
Rv0129c, Rv1886, Rv3804	Ag85 complex	1017.15	(F)SRPGLPVEY(L)
Rv3418c	GroES	1171.27	(W)DEDGEKRIPL(D)
Rv2101	HelZ	1394.53	(T)LEEKIDEM(ox)IEE(K)
Rv3417	GroEL1	1135.24	(A)KEIELEDPY(E)
Rv3763c	LpqH	1375.58	(T)GVDMANPM(ox)SPVNK(S)

**Table 3:** Peptides identified from MALDI-TOF analysis of fraction 9–7 of pronase digested WCL from the X4-19 ΔHspX Mtb strain.

Protein accession number	Protein name	Observed mass (M + H)	Peptide sequence
Rv1860	Mpt32	933.07	PPFPGQPPP
		1374.51	EPAPAPAPAGEVAPT
		1758.01	APPAPAPAPAEPAPAPAPA
Rv2560	Hypothetical protein Rv2560	1230.39	GPPPGPPPPGYPT
Rv0014c	PknB	2162.47	NPPANQTSAITNVVIIIVGSGP
Rv3448	EccD4	2164.69	PWIAALTAMLAAVAMLGFVA

### The amino terminus of native MPT32 contains the HLA-E epitope

**Figure 2:**
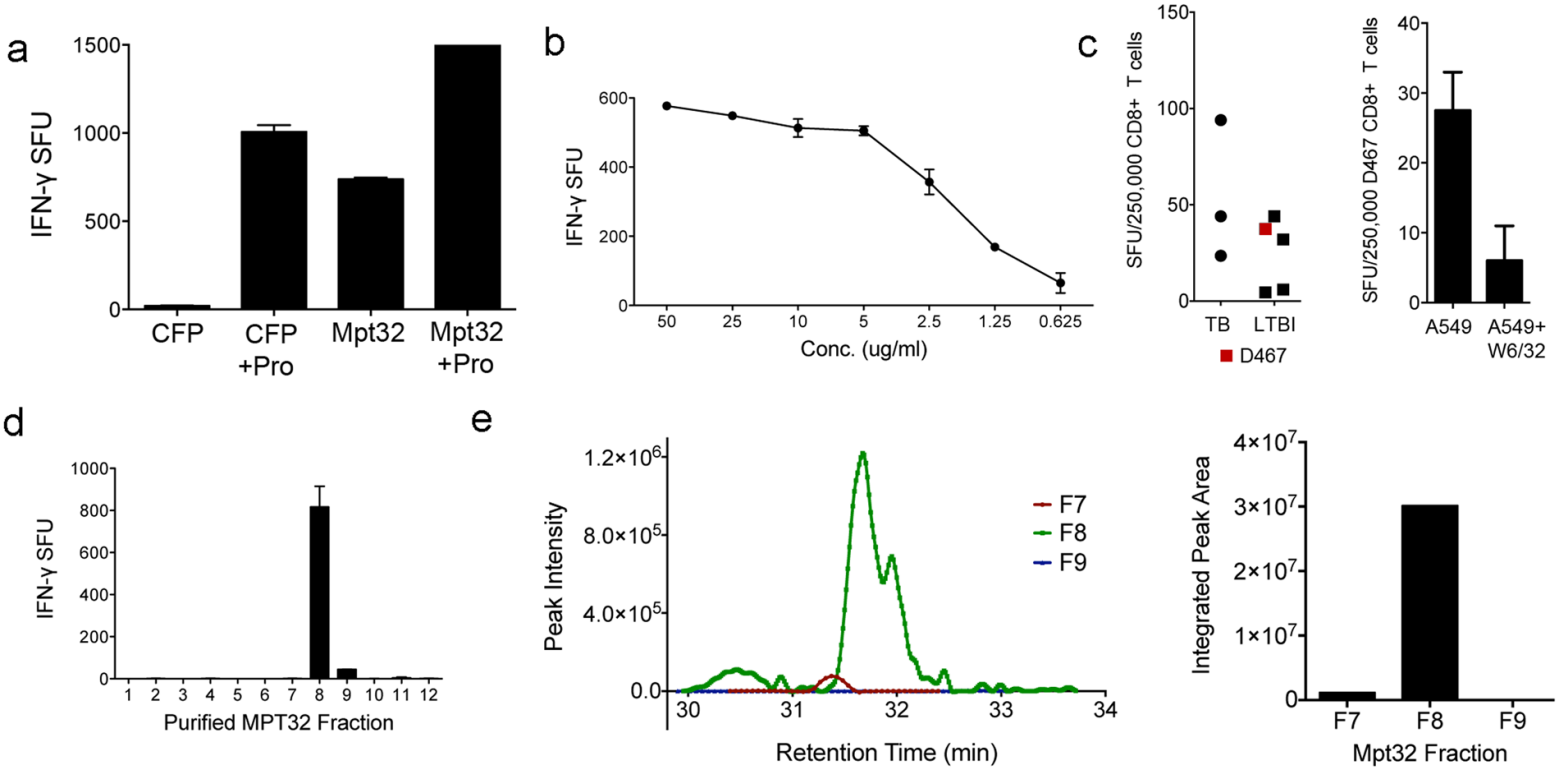
The amino terminus of native MPT32 contains the HLA-E epitope. (**a**,**b**) DC were pulsed with the following samples and used as antigen presenting cells in an ELISPOT assay with IFN-γ release (spot forming units (SFU)) by the HLA-E restricted T cell clone, D160 1–23, as a readout. (**a**) Culture filtrate protein (CFP) or native Mpt32 left untreated or pronase-digested. (**b**) A titration of pronase-digested native Mpt32. (**c**) Autologous DC were pulsed with pronase-digested MPT32 and used as antigen presenting cells in ELISPOT assay with IFN-γ release (spot forming units (SFU)) by purified CD8^+^ T cells from Mtb infected or latently infected donors (left). The pan Class I blocking antibody W6/32 was incubated with A549 cells for 1 hour prior to addition of MPT32. ELISPOT with purified CD8^+^ T cells from D467 was performed as described for DC (right). (**d**) DC were pulsed with RP-HPLC fractions of native Mpt32 and used as antigen presenting cells in an ELISPOT assay with IFN-γ release (spot forming units (SFU)) by the HLA-E restricted T cell clone, D160 1–23, as a readout. (**e**) The unique peptide signal in F8 of pronase-digested Mpt32 corresponds to pronase digestion product and putatitive N-terminal glycopeptide (n-APPAPAP[162.1]PVAPPPPAAA-c) observed as a double charged mass of 827.84 Da. Additional peptides and masses are listed in Supplemental Table 2.

MPT32, a 45 kDa alanine and proline-rich glycoprotein^21^, is a known CD4^+^^22^ and CD8^+^^23^ T cell antigen that is secreted into the culture filtrate of actively growing cells. To confirm that MPT32 contains the antigen for D160 1–23, native MPT32 was isolated from the culture filtrate proteins (CFP) of Mtb. Whole or pronase-digested native MPT32 and CFP were then used in an IFN-γ ELISPOT assay with D160 1–23. Pronase-digested MPT32 resulted in strong IFN-γ production by D160 1–23, as did pronase-digested CFP (Fig. 2a). Titration of purified pronase-digested native MPT32 demonstrated that T cell reactivity was observed down to a protein concentration of 0.625 ug/ml (Fig. 2b). To demonstrate that these findings are relevant to other Mtb infected (TB) or latently infected (LTBI) individuals we measured responses to autologous DC pulsed with pronase-digested MPT32. Here, we show that purified CD8^+^ T cells from 6 of 8 TB or LTBI donors contain a subset of cells that produce IFN-γ (Fig. 2c, left). We then used MPT32-pulsed A549 cells, an HLA-A, B, and C mismatched antigen presenting cell, and blocking with the pan-MHC Class I W6/32 antibody (Fig. 2c, right) with one of these donors (D467, highlighted in red) to demonstrate that it is highly likely that these responses are HLA-E restricted.

To identify the MPT32 antigen, the complexity of the source of biological activity in the native MPT32 protein was further reduced by performing RP-HPLC fractionation of pronase-digested native MPT32. The biological activity of MPT32 was primarily isolated to a single fraction, F8 (Fig. 2d). LC-MS analysis of fraction 8 identified a total of 10 unique peptides (normalized weighted spectra), with F8 containing approximately 20% of the MPT32 protein sequence. All unique peptide sequences identified in F8 are listed in Table 4. Tandem MS analyses to identify glycosylated MTP32 peptides did not yield convincing results, therefore predicted glycopeptide fragments belonging to the N-terminus of MPT32^21, 24^ were input into Skyline, an open-source application for building a number of targeted mass spectrometry assays^25^. Here, we used Skyline to build a quantitative method for MS1 data corresponding to known glycopeptides of MPT32 that are putatively generated following digestion of MPT32 with pronase (Supplemental Table 1). We then analyzed the MS1 mass spectrometry MS signal data from F7, 8, and 9 using this method and identified one MS1 signal uniquely abundant in F8, corresponding to the MPT32 n-terminal glycopeptide APPAPAT[+162.1]PVAPPPPAAA (Fig. 2e).

**Table 4:** Peptides identified from RP-HPLC analysis of fraction 8 of pronase digested native MPT32.

Sequence [Start, Stop]	Proba-bility	SEQU-EST XCorr	SEQU-EST ΔCn	Mascot ion score	Mascot identity score	Mascot Δ ion score	Obser-ved mass	Actual mass	Charge
(N)AQPGDPNAAPPPADPNAPPPPV(I) [81,102]	100%			114.9	71.27918	73.88	1,044.38	2,086.75	2
(A)APPPADPNAPPPPV(I) [89,102]	100%			91.28	71.347946	28.79	669.16	1,336.31	2
(A)APPPADPNAPPPPVIAPN(A) [89,106]	100%	3.6078	0.3418				578.73	1,733.17	3
(D)PNAPPPPVIAPN(A) [95,106]	98%			83.42	71.37691	19.68	592.73	1,183.45	2
(L)SKTTGDPPFPGQPPPVAN(D) [144,161]	100%			74.97	71.3026	40.03	903.99	1,805.97	2
(S)KTTGDPPFPGQPPPVAN(D) [145,161]	97%			70.91	71.33094	29.11	860.83	1,719.65	2
(K)TTGDPPFPGQPPPVAN(D) [146,161]	100%	2.1992	0.3939				1,591.83	1,590.82	1
(T)GDPPFPGQPPPVAN(D) [148,161]	100%			84.21	71.40511	27.44	695.76	1,389.51	2
(L)VAPPPAPAPAPAEPAPAPAPA(G) [287,307]	100%	3.8718	0.3647				620.15	1,857.43	3
(V)APPPAPAPAPAEPAPAPAPA(G) [288,307]	100%	3.821	0.1299				1,758.12	1,757.11	1

### Native MPT32 glycosylated peptides are antigenic

**Figure 3:**
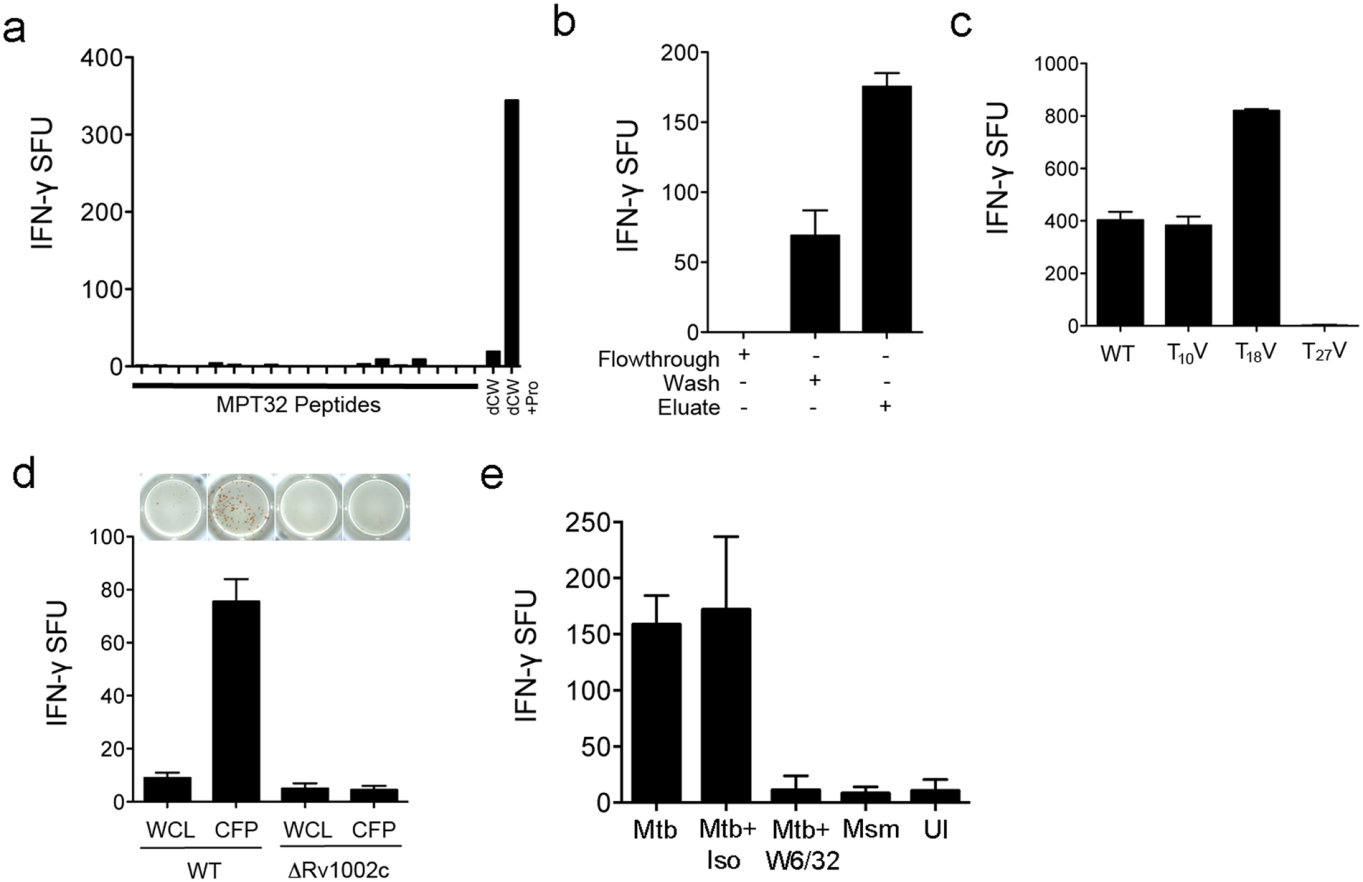
Native MPT32 glycosylated peptides are antigenic. (**a**–**d**) DC were pulsed with the following samples and used as antigen presenting cells in an ELISPOT assay with IFN-γ release (spot forming units (SFU)) by the HLA-E restricted T cell clone, D160 1–23, as a readout. (**a**) Synthetic overlapping peptides representing Mpt32. (**b**) Flow-through, wash, and eluate following concavalin-A column enrichment of pronase-digested native Mpt32. (**c**) Pronase-digested CFP from the Mpt32 T_10_V, T_18_V, and T_27_V recombinant Mtb strains. (**d**) Pronase digested whole cell lysates and culture filtrate proteins from wild-type Mtb or the ΔRv1002c Mtb mutant. (**e**) DC were infected with *M*. *smegmatis* (Msm) or Mtb as a positive control or left uninfected (UI) and used as antigen presenting cells in an ELISPOT assay with IFN-γ release (spot forming units (SFU)) by the HLA-E restricted T cell clone, D160 1–23, as a readout. Blocking with the W6/32 antibody is shown for Mtb infected cells. Results are the mean and standard deviation from four independent experiments.

We employed several methods to define the minimal epitope within the MPT32 protein. Testing of overlapping synthetic peptides representing the regions of MPT32 identified in F8 by ELISPOT assay with D160 1–23 did not result in any biological activity (Fig. 3a). Because some amino acids of the MPT32 sequences identified in the fraction are glycosylated, and overlapping synthetic peptides did not induce T cell responses, we used several methods to evaluate whether MPT32 glycosylation was required for T cell response. First, we enriched for glycopeptides present in the native MPT32 pronase digest using a Concavalin-A (ConA) lectin column. T cell activity was predominantly retained in the ConA column eluate containing mannosylated proteins (Fig. 3b), supporting the requirement for glycosylation. Next, we generated constructs to express recombinant MPT32 with point mutations at three mannosylated N-terminal threonines (T_10_V, T_18_V, and T_27_V) in a ΔMPT32 Mtb strain. Culture filtrate protein from the recombinant MPT32 strains were pronase digested and tested for T cell activity. Substitution of valine for threonine at position 10 (T_10_V) had no effect on T cell response, substitution of valine for threonine at position 18 (T_18_V) resulted in an increased T cell response, while substitution of valine for threonine at position 27 (T_27_V) completely abrogated the response (Fig. 3c). Finally, we tested Mtb with a complete deletion of the Mtb protein, ΔRv1002c, which encodes a mannosyltransferase that modifies proteins at serine and threonine residues with mannose^26^, including MPT32^27^. Analysis of the antigenic activity of pronase digested whole cell lysates and cell wall preparations from the ΔRv1002c mutant indicated that O-mannosylation is required for eliciting T cell responses (Fig. 3d). To validate these results, we tested for the ability of the D160 1–23 clone to recognize infection with *Mycobacterium smegmatis*, a non-pathogenic relative of Mtb that expresses a homolog of MPT32 (30% sequence homology at the N-terminus) that does not contain a threonine at positions 10, 18, or 27 (Fig. 3e). These results strongly support the conclusion that the epitope for the HLA-E restricted D160 1–23 clones includes the N-terminal T_27_ and that Mtb-specific O-linked mannosylation is required for HLA-E T cell activity.

## Discussion

CD8^+^ T cells play an important role in protective immunity against Mtb^28^. Classically-restricted Mtb-reactive CD8^+^ T cells recognize a highly diverse repertoire of peptides associated with classical class I molecules (HLA-Ia). Non-classical HLA-Ib molecules also recognize a variety of mycobacterial antigens, including glycolipids presented on CD1 molecules^29^ and vitamin B metabolites presented on MR1^2^, and protein-based ligands presented on HLA-E^13, 15^. Although a role for non-classical CD8^+^ T cells during Mtb infection is not yet clear, non-classically restricted CD8^+^ T cells make up a majority of Mtb-specific CD8^+^ T cell response^30^, suggesting they may be important to the immune response to Mtb infection. HLA-E has limited polymorphism^31^, and is likely to present a limited repertoire of antigens. Additionally, in contrast to classical class I molecules, HLA-E is not downregulated during HIV infection^32^. Together, these factors make targeting Mtb-specific HLA-E restricted CD8^+^ T cell responses a novel and attractive approach for vaccine development.

We previously isolated an Mtb-specific HLA-E restricted CD8^+^ T cell clone (D160 1–23) from a latently infected individual^13^, and sought to use this clone to identify Mtb ligands for HLA-E. Prior definition of D160 1–23 allowed for a biochemical and proteomic approach to identify the cognate ligand. Using these methods, we demonstrate that the N-terminal O-mannosylated glycopeptide of MPT32 contains the epitope for D160 1–23. O-mannosylation of serine or threonine residues of mycobacterial proteins has been described for T cell antigens including the 19 kDa lipoglycoprotein^33^ and the B cell antigen SodC^34^, however it is not known whether glycosylation of these proteins contributes to cognate interactions between the class I molecule and the T cell receptor. MHC-I molecules are capable of binding and presenting glycosylated peptides^35^, and presentation of aberrant glycosylated peptides is thought to be important to T cell recognition of tumor antigens^36^. This is the first description of a pathogen-derived glycosylated antigen recognized by HLA-E restricted CD8^+^ T cells. These findings may be important to vaccine and drug development as the enzymes responsible for glycosylation of MPT32 are specific to mycobacteria. In this regard, even between Mtb and the related non-pathogen *M. smegmatis*, there are differences in the degree of glycosylation of MPT32^37^. The potential importance of glycosylation to T cell recognition during Mtb infection is underscored by the inability of D160 1–23 to recognize *M. smegmatis*-infected target cells.

MPT32 is a relatively well-characterized secreted Mtb protein. MPT32 was first described by Nagai *et al*.^38^ in 1991 as a component of the Mtb secretome. Sites of MPT32 glycosylation were then thoroughly characterized by Dobos *et al*.^21, 24^, and Romain *et al*.^39^. In addition to stimulating antibody production^40^, MPT32 also strongly induces the production of IFN-γ by both CD4^+^ and CD8^+^ T cells^23, 39, 41^. Additionally, MPT32 provides protection in guinea pig challenge models when administered as DNA or protein subunit vaccine^23, 41^. Although MPT32 is not glycosylated when administered as a component of subunit vaccines, a number of groups have looked at the impact of glycosylation on antigenicity, immunogenicity, and protection. Romain *et al*. demonstrated that deglycosylation of MPT32 resulted in reduced DTH reaction in BCG immunized guinea pigs as well as reduced responses of CD4^+^ T cells in *ex vivo* stimulation assays^39^. Similarly, Horn *et al*. demonstrated that deglycosylation of MPT32 resulted in decreased stimulation of T cells isolated from the lymph nodes of BCG vaccinated guinea pigs^37^. More recently, Nandakumar *et al*. demonstrated that while glycosylation of MPT32 was important to antigenicity, it was dispensable for immunogenicity and protection in a mouse challenge model^42^. We have demonstrated that human HLA-E restricted T cells recognize a glycosylated MPT32 peptide, indicating that glycosylation may be important in human vaccination studies. However, further studies are required to determine whether HLA-E restricted responses to glycosylated Mtb proteins can be harnessed to elicit protection.

In our study, we used IFN-γ production by the HLA-E restricted CD8^+^ T cell clone to measure T cell activation. IFN-γ is a Th1 cytokine thought to play an essential role in protection during Mtb infection^43, 44^. The role of Th2 cytokines in mycobacterial infection is not clear, however recent studies have identified a new population of HLA-E-restricted Th2 cytokine producing CD8^+^ T cells^15, 17, 18^. In these studies, putative HLA-E binding peptides were identified through bioinformatic analysis of the Mtb genome. Tetramers were generated with the peptides and HLA-E restricted T cells recognizing the peptides were detected among circulating T cells. When isolated from human PBMC and stimulated with peptide, these HLA-E restricted T cells produce Th2 cytokines. These results reveal a novel Th2 T cell population in the human immune response to Mtb. The role of glycosylated epitopes, such as the MPT32 peptide identified here, in production of Th1 or Th2 cytokines by HLA-E restricted CD8^+^ T cells remains to be explored, but these results demonstrate that HLA-E restricted CD8^+^ T cells have the potential to play a unique and important role in the human immune response to infection with Mtb.

## Materials and Methods

### Human Subjects and ethics statement

This study was conducted according to the principles expressed in the Declaration of Helsinki. All study participants were adults. Study participants, protocols, and consent forms were approved by the Oregon Health & Science University Institutional Review Board (OHSU IRB0000186). Written informed consent was obtained from all participants and all samples were anonymized using a random three digit code by the study coordinator.

Peripheral blood mononuclear cells (PBMC) were obtained by apheresis from healthy, LTBI, and Mtb-infected adult donors recruited from OHSU as previously described^45^, or via IRB-approved advertisement at local TB clinics. Uninfected individuals were defined as healthy individuals with a negative tuberculin skin test and no known risk factors for infection with Mtb. Individuals with LTBI were defined as healthy individuals with a positive tuberculin skin test and no symptoms and signs of active TB. Individuals with active TB were diagnosed by the TB Controller for Multnomah or Washington Counties in Oregon, United States, and confirmed by positive sputum culture for Mtb.

### Bacteria and Cell Lines

*Mycobacterium tuberculosis* strain H_37_Rv was originally provided as strain TMC102 from the Trudeau Mycobacterial Collection and is currently available from the Biodefense and Emerging Infections Resources Repository (BEI Resources, Manassas, VA). Similarly, CDC1551 was originally provided from the Centers for Disease Control and Prevention and is available from BEI Resources. X4-19 (ΔHspX) was kindly provided from Russel Karls and Fred Quinn (University of Athens, GA).

A549 cells were obtained from the American Type Culture Collection (ATCC, CCL-185) and cultured in F12K with 10% heat inactivated FBS. Human monocyte-derived dendritic cells (DC) were prepared as described^46^. Briefly, PBMC obtained as described above were resuspended in RPMI with 2% heat-inactivated human serum (HuS) and allowed to adhere to a T-75 (Costar) flask at 37C for 1 hr. After gentle rocking, non-adherent cells were removed and 10% heat-inactivated HuS in RPMI containing 10 ng/ml IL-4 (R&D Systems) and 30 ng/ml GM-CSF (Sanofi) was added to the adherent cells. After 5 days, cells were harvested with cell-dissociation medium (Sigma-Aldrich) and used as indicated in assays. The D160 1–23 human HLA-E restricted CD8^+^ T cell clone was expanded and maintained as previously described^13^.

### Isolation of Subcellular Fractions and Delipidation of Cell Wall

*M. tuberculosis*, H_37_Rv was cultured in glycerol-alanine-salts (GAS) media for 14 days at 37 °C, followed by harvest of supernatant and cells, lysis and subcellular fractionation as described previously^47^. Briefly, culture filtrate proteins were separated from cells via filtration through a 0.22μm membrane and concentrated using a stirred cell apparatus under N_2_, followed by dialysis into 0.01 M NH_4_HCO_3_. Whole cells were inactivated by γ-irradiation and resuspended in breaking buffer (PBS, 1 mM EDTA) supplemented with protease inhibitors, DNase and RNase for serial passage through a French pressure cell (Thermo Scientific). Whole cell lysate was dialyzed into 0.01 M NH_4_HCO_3_, and centrifuged at 27,000× g at 4 °C for 1 h to generate cell wall. The supernatant was subsequently centrifuged at 100,000× g at 4 °C for 4 h to recover cell membrane. Cell wall, membrane and cytosol were all dialyzed and protein quantified via BCA (Thermo Pierce).

The cell wall fraction was subjected to organic extraction to remove non-covalently associated lipids and glycolipids^48^. Lyophilized cell wall was subjected to two extractions of 2 h each followed by one 18 h extraction with chloroform/methanol (2:1, v/v) at a ratio of 30 mL/g of cell wall. Extractions were performed at 22 °C with agitation. Centrifugation at 27,000 g for 30 min was performed to collect cell wall material. The 2:1 extracted cell wall was dried under N2 and further extracted twice for 2 h and one 18 h extraction with chloroform/methanol/water (10:10:3, v/v/v) each at 22 °C. The fully delipidated cell wall was dried under N_2_ and resuspended in PBS, 0.1% ASB-14 pH 7.4 to maximize solubility. Cell wall protein was quantified by bicinchoninic acid (BCA) assay (Thermo Pierce).

### Purification of Native MPT32

Ammonium sulfate (NH_4_)_2_SO_2_ was added to the culture filtrate proteins (CFP) to a final concentration of 40% and proteins allowed to separate for 1 hour at 4 °C. The CFP was then centrifuged at 16,000× g for 30 minutes and supernatant removed for a second salt cut at 70%. The 40% pellet was resuspended in phenyl sepharose buffer A (10 mM KH2PO4 (pH 7.2), 1 mM EDTA, 1 mM DTT (1 L) and a gradient of buffer B (10 mM Tris-Base (pH 8.9), 1 mM EDTA, 1 mM DTT (1 L), then C (10 mM Tris-Base (pH 8.9), 1 mM EDTA, 1 mM DTT, 50% ethylene glycol (v/v)) applied to separate proteins. Unbound material from the phen-seph column (flow through) was exchanged into concavalin-A (ConA) binding buffer (50 mM KH_2_PO_4_, 500 mM NaCl, 1 mM each of MgCl_2_, CaCl_2_, MnCl_2_ and 1 mM DTT) and applied to a ConA column. Bound protein was eluted with excess 1 M Methyl α-D-mannopyranoside. MPT32 was polished over a C18 reverse phase column in 20 mM ammonium bicarbonate, 1 mM DTT and eluted off with an increasing gradient of 20 mM ammonium bicarbonate, 70% acetonitrile.

### Pronase Digestion

Pronase (250 ku, EMD Millipore) was resuspended in digestion buffer - PBS, 0.1% ASB-14 for subcellular fractions or 0.2 M NH_4_HCO_3_ for purified proteins, at a concentration of 5.0 mg/ml. Digestion was performed at 37 °C, 16 h at an enzyme to substrate ratio of 1:20 for all samples. Enzymatic reactivity was quenched with rapid freeze thaw cycles at −80 °C.

### IFN-γ Elispot assay

IFN-γ ELISPOT analysis was performed as previously described^13^. Briefly, 96-well nitrocellulose backed plates were coated as recommended by the manufacturer with capture mouse anti-IFN-γ overnight at 4 °C. Plates were then washed three times with PBS (Invitrogen) and blocked with RPMI + 10% HS for 1 h at room temperature. Antigen presenting cells (20,000) were added, then pulsed with antigen for 1 hr. T cell clones (20,000) were then added, and the plate incubated overnight at 37 °C. Plates were extensively washed and anti-IFN-γ secondary antibody conjugated to HRP was added. AEC developer substrate was added and the reaction stopped by washing with distilled water.

### Reverse Phase HPLC

Whole protein digests (1 mg to 5 mg) were separated on Waters Alliance 2695 HPLC (Waters Inc, Millford) using a Grace Vydac Everest 300 Å monomeric C18 column (4.6 × 150 mm, 238EV5115). Fractions were collected every 2 minutes over a gradient of 0–50% B over 30 minutes, 0.5 ml/min. Buffer A − 0.1% TFA in water, Buffer B − 0.1% TFA, 90% acetonitrile. For fraction 9 A, the gradient was modified to begin at 15% B and increased to 30% B over 30 minutes. Fractions were collected every 3 minutes at a flow rate of 0.5 ml/min. For all peptide separations fractions were concentrated using vacuum centrifugation and resuspended at an approximate concentration of 0.1 ug/ul in Buffer A. Sample were kept frozen at −80 °C prior to ELISPOT testing.

### Concavalin-A lectin chromatography of peptides

3 mg of purified MPT32 was subjected to Pronase digestion as described above (concentration of 1 mg/ml). Peptides were incubated with in ConA buffer as described above with rocking, in the presence of 2 ml of Con-A conjugated sepharose slurry (i.e. 1 ml settled resin) (GE Life Sciences) at 4 °C, for 16 h in a 15 ml reaction tube. Resin-peptide mix was poured into an open column and allowed to settle. Flow through was collected. Resin was washed with 10 column volumes of binding buffer and collected. The peptides were eluted off with 10 CV of elution buffer. Collected fractions were exchanged back into 0.1% TFA after processing through solid-phase sep-pak columns (Waters) following manufacturer’s instructions.

### Mass spectrometry

Liquid Chromatography Mass Spectrometry (LC-MS): Peptides were separated on a nanospray column (Zorbax C18, 5 μm, 75 μm ID 6 150 mm column) and samples were eluted into a LTQ linear ion trap mass spectrometer (Thermo) as described previously^49^. MALDI-TOF: 1 ml of peptide sample is mixed with 1 ml of a mixed matrix solution (10 mg/ml α-Cyano-4-hydroxycinnamic acid (CHCA) and 10 mg/ml 2,5-Dihydroxybenzoic acid (DHB); 1:1 v/v) in 50% ACN, 0.1% TFA. The mixture was spotted on the MALDI target and allowed to air dry. The sample was analyzed by an Ultraflex-TOF/TOF mass spectrometer (Bruker Daltonics, Billerica) in positive ion, reflector mode using a 25 kV accelerating voltage. External calibration was performed using a peptide calibration mixture (4 to 6 peptides) on a spot adjacent to the sample. The raw data was processed in the FlexAnalysis software (version 3.3, Bruker Daltonics).

### Peptide and Protein Identification

LC-MS Database Searching: Tandem mass spectra were extracted, charge state deconvoluted and deisotoped by Xcalibur version 1.7 SP2. All MS/MS samples were analyzed using Sequest (Thermo Fisher Scientific, v.27, rev. 11) and MASCOT (Matrix Science, v.2.3). Sequest and MASCOT were set up to search the MtbV3_Reverse database (7992 entries) assuming non-specific cleavage. Parameters for both search engines were set to a fragment ion mass tolerance of 1.5 Da and a parent ion tolerance of 3.0 Da. Oxidation of methionine was specified as variable modification. For glycopeptides, a modification of mannose (+162 da) and mannobiose (+324) were set as variable modification on threonine. All mass spectrometry proteomics data have been deposited to the ProteomeXchange Consortium via the PRIDE partner repository^50, 51^ with the dataset identifier PXD005765 and 10.6019/PXD005765.

MALDI-TOF spectra were collected and select ions were chosen for fragmentation. Peptide sequences were elucidated via *de novo* calculation of amino acid fragments. M + H single charged ion masses were matched if identified using LC-MS protein identification. Scaffold (version Scaffold_3.5.1, Proteome Software Inc.) was used to validate MS/MS based peptide and protein identifications. Peptide identifications were accepted if they exceeded specific database search engine thresholds. Sequest identifications required at least deltaCn scores of greater than 0.2 and XCorr scores of greater than 1.8, 2.0, 3.0 and 4.0 for singly, doubly, triply and quadruply charged peptides. Protein identifications assigned within each peptide fraction were accepted if they contained at least 2 identified peptides. All peptide spectra were manually inspected for sequence coverage and signal to noise. MS1 peak detection of tandem LC-MS data was performed in Skyline (MacCoss Lab Software, v.3.6). Non-specific cleavage products resulting from pronase digestion of MPT32 were predicted and listed as Precursor Mass targets (+2 and +3 charge states). Putative target peptides were considered only if the M, M +1, and M +2 isotope peak signal was 3X above background and isotope dot products (idotp) ratios of 0.9 or greater.

### Generation of T10V, T18V, and T27V MPT32 mutants

Each of the 3 N-terminal threonine (Thr) sites of glycosylation were subjected to site-directed mutagenesis (Thr −>Val) (QuickChange II XL, Agilent). Briefly, the plasmid pMV261 containing the MPT32 gene was amplified with mutagenesis primers: T10V (5′-gcccccggtacccgtaacggccgcctcg-3′), T18V (5′tcgccgccgtcggtcgctgcagcgcc-3′), and T27V (5′-acccgcaccggcggtacctgttgccccc-3′). The mutants (T10V, T18V, T27V) were transformed into an Mpt32 knock-out strain of Mtb, CDC1551 (kindly provided by Dr. Gyanu Lamichane at Johns Hopkins University). Transformed cells were grown for two weeks in glycerol, alanine, salts medium and cells harvested from the culture filtrate (CFP). Purification of wild type Mpt32 from Mtb CDC1551 was conducted similarly as above with minor modifications. Briefly, Mtb CFP was concentrated using a Pellicon system with a 10 kDa membrane (Millipore) and proteins precipitated with 40% ammonium sulfate. The precipitate was enriched for glycoproteins and glycoconjugates over a concavalin-A lectin chromatography column (as above), and final purification of MPT32 was achieved by resolving the ConA column eluate using C4 RP-HPLC (using the same conditions as above for C18 polishing).

### Disruption of *Rv1002c* in *M. tuberculosis* by allelic replacement

The avirulent auxotrophic *M. tuberculosis* H37Rv strain mc^2^6206 (Δ*panCD* Δ*leuCD*) (kindly provided by Dr. W. R. Jacobs, Albert Einstein College of Medicine) was grown at 37 °C in Middlebrook 7H9-OADC-0.05% tyloxapol supplemented with 0.2% casaminoacids, 48 μg/ml pantothenate and 50 μg/ml L-leucine or on similarly supplemented Middlebrook 7H11-OADC agar medium. When required, kanamycin (Kan) and hygromycin (Hyg) were used at concentrations of 25 and 50 mg/ml, respectively.

The *Rv1002c* locus of *M. tuberculosis* mc^2^6206 was disrupted by recombineering using plasmid pJV53 to transiently express the highly active mycobacteriophage-encoded recombinases, gp60 and gp61, as described^52^. The linear DNA substrate used to achieve allelic replacement at the *Rv1002c* locus was PCR-amplified from *M. tuberculosis* H37Rv genomic DNA using primers Rv1002c-KOF1 (5′-CGGGTAAACACGTCACCGA-3′) and Rv1002c-KOR1 (5′-ACGATGGTCGACACCGATAC-3′), and 1,390-bp of the *Rv1002c* sequence flanked by two AgeI sites was replaced by a hygromycin resistance gene. *M. tuberculosis* mc^2^6206 electrocompetent cells expressing the recombinases from pJV53 were transformed with the linear DNA substrate and double cross-over mutants selected on 7H11-OADC-Hyg plates at 37 °C. Allelic replacement at the *Rv1002c* locus was confirmed by PCR and sequencing.

### Data Availability

All data generated or analyzed during this study (except raw mass spectrometry data, see below) are included in the published article. All mass spectrometry proteomics data have been deposited to the ProteomeXchange Consortium via the PRIDE partner repository^50, 51^ with the dataset identifier PXD005765 and 10.6019/PXD005765.

## Electronic supplementary material

Supplementary Information
