# Supplementary material for: HLA-E Presents Glycopeptides from the Mycobacterium tuberculosis Protein MPT32 to Human CD8+ T cells
Source: Sci Rep. 2017 Jul 4;7:4622. doi: 10.1038/s41598-017-04894-0 (PMC5496856; doi:10.1038/s41598-017-04894-0)
Supplement: Supplementary file 1 — Supplementary Information [file 41598_2017_4894_MOESM1_ESM.pdf]

Supplementary Dataset

HLA-E Presents Glycopeptides from the *Mycobacterium tuberculosis* Protein MPT32 to Human CD8$^+$ T cells

Melanie J. Harriff, Lisa M. Wolfe, Gwendolyn Swarbrick, Megan Null, Meghan E. Cansler, Elizabeth T. Canfield, Todd Vogt, Katelynn Gardner Toren, Wei Li, Mary Jackson, Deborah A. Lewinsohn, Karen M. Dobos, David M. Lewinsohn.

Supplemental Table 1. Skyline analysis of putative MPT32 glycopeptides generated following pronase digestion.

Replicate Name	Precursor	Modified Sequence	Precursor Charge	Isotope Dot Product	Precursor Neutral Mass	Precursor Mz	Precursor Results Summary
MPT32_pronase_Fract 7	668.8484++	APPPADPNAPPPPV	2	0.9979	1335.682302	668.848427	RT: 31.46+/-0.52 Area: 7440955127+/-8318260587
MPT32_pronase_Fract 7	446.2347+++	APPPADPNAPPPPV	3	0.9912	1335.682302	446.23471	RT: 31.48+/-0.46 Area: 885070487+/-992422562
MPT32_pronase_Fract 7	809.4354++	APPPADPNAPPPPVIAP	2	0.9758	1616.856243	809.435398	RT: 39.49+/-1.39 Area: 173165888+/-276506081
MPT32_pronase_Fract 7	539.9594+++	APPPADPNAPPPPVIAP	3	0.9711	1616.856243	539.959357	RT: 39.54+/-1.4 Area: 54326258+/-87496050
MPT32_pronase_Fract 7	547.7725++	APDAGPPQRW	2	0.9652	1093.530492	547.772522	RT: 31.3+/-7.78 Area: 147872961+/-151495169
MPT32_pronase_Fract 7	553.2693++	GDPPFPGQPPP	2	0.959	1104.52401	553.269281	RT: 33.21+/-5.05 Area: 227793374+/-370828809
MPT32_pronase_Fract 7	638.3220++	GDPPFPGQPPPVA	2	0.9562	1274.629538	638.322045	RT: 36.78+/-8.33 Area: 472843861+/-772449774
MPT32_pronase_Fract 7	881.4314++	GDPPFPGQPPPVANDTR	2	0.9543	1760.848198	881.431375	RT: 30.48+/-10.48 Area: 27434791+/-46926459
MPT32_pronase_Fract 7	438.7323++	PGQPPPVAN	2	0.9409	875.450117	438.732334	RT: 16.72+/-0.07 Area: 9466569+/-12059698
MPT32_pronase_Fract 7	425.8838+++	GDPPFPGQPPPVA	3	0.9372	1274.629538	425.883789	RT: 37.07+/-8.64 Area: 295920+/-184883
MPT32_pronase_Fract 7	1044.2071+++	AQPGDPNAAPPPADPNAPPPPVIAPNAPQPVR	3	0.871	3129.599582	1044.207137	RT: 72.49+/-34.7 Area: 733137+/-1223125
MPT32_pronase_Fract 7	587.9567+++	GDPPFPGQPPPVANDTR	3	0.8618	1760.848198	587.956675	RT: 30.51+/-10.46 Area: 10508742+/-11753091
MPT32_pronase_Fract 7	879.4647++	APPPAPAPAPAEPAPAPAPA	2	0.8588	1756.914821	879.464686	RT: 32.44+/-0.59 Area: 14245392103+/-24539114849
MPT32_pronase_Fract 7	866.4569++	APPPADPNAPPPPVIAPN	2	0.8382	1730.899171	866.456861	RT: 47.97+/-18.87 Area: 3674837682+/-5846015549
MPT32_pronase_Fract 7	739.3697++	TTGDPPFPGQPPPVA	2	0.8359	1476.724895	739.369723	RT: 33.77+/-7.11 Area: 259280409+/-444539704
MPT32_pronase_Fract 7	609.3193++	PPFPGQPPPVAN	2	0.8239	1216.624058	609.319305	RT: 34.5+/-4.73 Area: 1692571500+/-2745484031
MPT32_pronase_Fract 7	746.9116++	APPAPATPVAPPPPAAA	2	0.8155	1491.808565	746.911558	RT: 38.24+/-9.25 Area: 1122217+/-1096919
MPT32_pronase_Fract 7	860.4387++	KTTGDPPFPGQPPPVAN	2	0.7779	1718.862785	860.438669	RT: 31.58+/-4.91 Area: 23776785+/-32629110
MPT32_pronase_Fract 7	796.3912++	TTGDPPFPGQPPPVAN	2	0.7373	1590.767822	796.391187	RT: 35+/-3.98 Area: 308608201+/-500464708
MPT32_pronase_Fract 7	695.3435++	GDPPFPGQPPPVAN	2	0.6831	1388.672465	695.343509	RT: 34.89+/-6.1 Area: 5539088265+/-8629471564
MPT32_pronase_Fract 7	696.3446+++	AQPGDPNAAPPPADPNAPPPPV	3	0.6426	2086.011969	696.344599	RT: 35.65+/-0.28 Area: 85089401+/-145989096
MPT32_pronase_Fract 7	459.5663+++	TGDPPFPGQPPPVA	3	0.6361	1375.677216	459.566348	RT: 34.57+/-6.3 Area: 107552+/-164500
MPT32_pronase_Fract 7	586.6455+++	APPPAPAPAPAEPAPAPAPA	3	0.6192	1756.914821	586.64555	RT: 32.57+/-0.45 Area: 6174432920+/-10641687371
MPT32_pronase_Fract 7	531.2632+++	TTGDPPFPGQPPPVAN	3	0.6082	1590.767822	531.263217	RT: 35.24+/-3.85 Area: 3798368+/-4759463
MPT32_pronase_Fract 7	592.3271++	PNAPPPPVIAPN	2	0.5858	1182.639708	592.32713	RT: 32.88+/-0.61 Area: 121712224+/-182266215
MPT32_pronase_Fract 7	803.3808++	GDPPFPGQPPPVANDT	2	0.5819	1604.747087	803.380819	RT: 38.78+/-4.97 Area: 379287309+/-296331013
MPT32_pronase_Fract 7	688.8459++	TGDPPFPGQPPPVA	2	0.5677	1375.677216	688.845884	RT: 34.65+/-6.73 Area: 54657021+/-67935056
MPT32_pronase_Fract 7	493.2489+++	TTGDPPFPGQPPPVA	3	0.5663	1476.724895	493.248908	RT: 33.65+/-7.01 Area: 205289+/-62379
MPT32_pronase_Fract 7	463.8981+++	GDPPFPGQPPPVAN	3	0.5518	1388.672465	463.898098	RT: 35+/-6.23 Area: 11532231+/-18190265
MPT32_pronase_Fract 7	577.9737+++	APPPADPNAPPPPVIAPN	3	0.548	1730.899171	577.973666	RT: 47.95+/-18.97 Area: 1866456883+/-2946169666
MPT32_pronase_Fract 7	535.9230+++	GDPPFPGQPPPVANDT	3	0.5261	1604.747087	535.922972	RT: 38.84+/-4.74 Area: 349117+/-415574
MPT32_pronase_Fract 7	1044.0133++	AQPGDPNAAPPPADPNAPPPPV	2	0.5215	2086.011969	1044.01326	RT: 35.64+/-0.28 Area: 54589894+/-93480742
MPT32_pronase_Fract 7	827.9380++	APPAPAT[+162.1]PVAPPPPAAA	2	0.518	1653.861389	827.93797	RT: 31.46+/-0.21 Area: 10426544+/-17078471
MPT32_pronase_Fract 7	903.9547++	SKTTGDPPFPGQPPPVAN	2	0.4678	1805.894814	903.954683	RT: 33.57+/-5.56 Area: 22846997+/-14405011
MPT32_pronase_Fract 7	745.8673++	TGDPPFPGQPPPVAN	2	0.4422	1489.720144	745.867348	RT: 35.18+/-5.92 Area: 658886112+/-879683890
MPT32_pronase_Fract 7	928.9989++	VAPPPAPAPAPAEPAPAPAPA	2	0.4343	1855.983235	928.998893	RT: 31.62+/-6.33 Area: 41490535+/-67333835
MPT32_pronase_Fract 7	602.9722+++	SKTTGDPPFPGQPPPVAN	3	0.4306	1805.894814	602.972214	RT: 33.67+/-5.45 Area: 9647879+/-9313381
MPT32_pronase_Fract 7	573.9615+++	KTTGDPPFPGQPPPVAN	3	0.4287	1718.862785	573.961538	RT: 31.58+/-5 Area: 9439565+/-10311970
MPT32_pronase_Fract 7	497.5807+++	TGDPPFPGQPPPVAN	3	0.3824	1489.720144	497.580657	RT: 35.25+/-5.8 Area: 634243+/-769151
MPT32_pronase_Fract 7	619.6684+++	VAPPPAPAPAPAEPAPAPAPA	3	0.3161	1855.983235	619.668354	RT: 31.61+/-6.32 Area: 70629415+/-106074768
MPT32_pronase_Fract 7	406.5486+++	PPFPGQPPPVAN	3	0.2434	1216.624058	406.548629	RT: 34.58+/-4.43 Area: 8739886+/-14571854
MPT32_pronase_Fract 8	586.6455+++	APPPAPAPAPAEPAPAPAPA	3	0.9989	1756.914821	586.64555	RT: 32.57+/-0.45 Area: 6174432920+/-10641687371
MPT32_pronase_Fract 8	879.4647++	APPPAPAPAPAEPAPAPAPA	2	0.9981	1756.914821	879.464686	RT: 32.44+/-0.59 Area: 14245392103+/-24539114849
MPT32_pronase_Fract 8	577.9737+++	APPPADPNAPPPPVIAPN	3	0.9977	1730.899171	577.973666	RT: 47.95+/-18.97 Area: 1866456883+/-2946169666
MPT32_pronase_Fract 8	695.3435++	GDPPFPGQPPPVAN	2	0.9975	1388.672465	695.343509	RT: 34.89+/-6.1 Area: 5539088265+/-8629471564
MPT32_pronase_Fract 8	668.8484++	APPPADPNAPPPPV	2	0.9966	1335.682302	668.848427	RT: 31.46+/-0.52 Area: 7440955127+/-8318260587
MPT32_pronase_Fract 8	803.3808++	GDPPFPGQPPPVANDT	2	0.9963	1604.747087	803.380819	RT: 38.78+/-4.97 Area: 379287309+/-296331013
MPT32_pronase_Fract 8	619.6684+++	VAPPPAPAPAPAEPAPAPAPA	3	0.9952	1855.983235	619.668354	RT: 31.61+/-6.32 Area: 70629415+/-106074768
MPT32_pronase_Fract 8	866.4569++	APPPADPNAPPPPVIAPN	2	0.9949	1730.899171	866.456861	RT: 47.97+/-18.87 Area: 3674837682+/-5846015549
MPT32_pronase_Fract 8	609.3193++	PPFPGQPPPVAN	2	0.9929	1216.624058	609.319305	RT: 34.5+/-4.73 Area: 1692571500+/-2745484031
MPT32_pronase_Fract 8	696.3446+++	AQPGDPNAAPPPADPNAPPPPV	3	0.9927	2086.011969	696.344599	RT: 35.65+/-0.28 Area: 85089401+/-145989096
MPT32_pronase_Fract 8	446.2347+++	APPPADPNAPPPPV	3	0.9918	1335.682302	446.23471	RT: 31.48+/-0.46 Area: 885070487+/-992422562
MPT32_pronase_Fract 8	928.9989++	VAPPPAPAPAPAEPAPAPAPA	2	0.9882	1855.983235	928.998893	RT: 31.62+/-6.33 Area: 41490535+/-67333835

MPT32_pronase_Fract 8	745.8673++	TGDPPFPGQPPPVAN	2	0.9873	1489.720144	745.867348	RT: 35.18+/-5.92 Area: 658886112+/-879683890
MPT32_pronase_Fract 8	547.7725++	APDAGPPQRW	2	0.9859	1093.530492	547.772522	RT: 31.3+/-7.78 Area: 147872961+/-151495169
MPT32_pronase_Fract 8	1044.0133++	AQPGDPNAAPPPADPNAPPPPV	2	0.9829	2086.011969	1044.01326	RT: 35.64+/-0.28 Area: 54589894+/-93480742
MPT32_pronase_Fract 8	860.4387++	KTTGDPPFPGQPPPVAN	2	0.9813	1718.862785	860.438669	RT: 31.58+/-4.91 Area: 23776785+/-32629110
MPT32_pronase_Fract 8	827.9380++	APPAPAT[+162.1]PVAPPPPAAA	2	0.9784	1653.861389	827.93797	RT: 31.46+/-0.21 Area: 10426544+/-17078471
MPT32_pronase_Fract 8	406.5486+++	PPFPGQPPPVAN	3	0.9759	1216.624058	406.548629	RT: 34.58+/-4.43 Area: 8739886+/-14571854
MPT32_pronase_Fract 8	553.2693++	GDPPFPGQPPP	2	0.9728	1104.52401	553.269281	RT: 33.21+/-5.05 Area: 227793374+/-370828809
MPT32_pronase_Fract 8	438.7323++	PGQPPPVAN	2	0.9712	875.450117	438.732334	RT: 16.72+/-0.07 Area: 9466569+/-12059698
MPT32_pronase_Fract 8	638.3220++	GDPPFPGQPPPVA	2	0.9694	1274.629538	638.322045	RT: 36.78+/-8.33 Area: 472843861+/-772449774
MPT32_pronase_Fract 8	463.8981+++	GDPPFPGQPPPVAN	3	0.963	1388.672465	463.898098	RT: 35+/-6.23 Area: 11532231+/-18190265
MPT32_pronase_Fract 8	497.5807+++	TGDPPFPGQPPPVAN	3	0.9601	1489.720144	497.580657	RT: 35.25+/-5.8 Area: 634243+/-769151
MPT32_pronase_Fract 8	592.3271++	PNAPPPPVIAPN	2	0.9557	1182.639708	592.32713	RT: 32.88+/-0.61 Area: 121712224+/-182266215
MPT32_pronase_Fract 8	539.9594+++	APPPADPNAPPPPVIAP	3	0.9489	1616.856243	539.959357	RT: 39.54+/-1.4 Area: 54326258+/-87496050
MPT32_pronase_Fract 8	573.9615+++	KTTGDPPFPGQPPPVAN	3	0.9386	1718.862785	573.961538	RT: 31.58+/-5 Area: 9439565+/-10311970
MPT32_pronase_Fract 8	425.8838+++	GDPPFPGQPPPVA	3	0.919	1274.629538	425.883789	RT: 37.07+/-8.64 Area: 295920+/-184883
MPT32_pronase_Fract 8	688.8459++	TGDPPFPGQPPPVA	2	0.915	1375.677216	688.845884	RT: 34.65+/-6.73 Area: 54657021+/-67935056
MPT32_pronase_Fract 8	531.2632+++	TTGDPPFPGQPPPVAN	3	0.9093	1590.767822	531.263217	RT: 35.24+/-3.85 Area: 3798368+/-4759463
MPT32_pronase_Fract 8	535.9230+++	GDPPFPGQPPPVANDT	3	0.9025	1604.747087	535.922972	RT: 38.84+/-4.74 Area: 349117+/-415574
MPT32_pronase_Fract 8	796.3912++	TTGDPPFPGQPPPVAN	2	0.8918	1590.767822	796.391187	RT: 35+/-3.98 Area: 308608201+/-500464708
MPT32_pronase_Fract 8	809.4354++	APPPADPNAPPPPVIAP	2	0.8565	1616.856243	809.435398	RT: 39.49+/-1.39 Area: 173165888+/-276506081
MPT32_pronase_Fract 8	602.9722+++	SKTTGDPPFPGQPPPVAN	3	0.8314	1805.894814	602.972214	RT: 33.67+/-5.45 Area: 9647879+/-9313381
MPT32_pronase_Fract 8	746.9116++	APPAPATPVAPPPPAAA	2	0.801	1491.808565	746.911558	RT: 38.24+/-9.25 Area: 1122217+/-1096919
MPT32_pronase_Fract 8	493.2489+++	TTGDPPFPGQPPPVA	3	0.7706	1476.724895	493.248908	RT: 33.65+/-7.01 Area: 205289+/-62379
MPT32_pronase_Fract 8	903.9547++	SKTTGDPPFPGQPPPVAN	2	0.758	1805.894814	903.954683	RT: 33.57+/-5.56 Area: 22846997+/-14405011
MPT32_pronase_Fract 8	881.4314++	GDPPFPGQPPPVANDTR	2	0.6629	1760.848198	881.431375	RT: 30.48+/-10.48 Area: 27434791+/-46926459
MPT32_pronase_Fract 8	587.9567+++	GDPPFPGQPPPVANDTR	3	0.6284	1760.848198	587.956675	RT: 30.51+/-10.46 Area: 10508742+/-11753091
MPT32_pronase_Fract 8	739.3697++	TTGDPPFPGQPPPVA	2	0.5188	1476.724895	739.369723	RT: 33.77+/-7.11 Area: 259280409+/-444539704
MPT32_pronase_Fract 8	1044.2071+++	AQPGDPNAAPPPADPNAPPPPVIAPNAPQPVR	3	0.5184	3129.599582	1044.207137	RT: 72.49+/-34.7 Area: 733137+/-1223125
MPT32_pronase_Fract 8	459.5663+++	TGDPPFPGQPPPVA	3	0.2614	1375.677216	459.566348	RT: 34.57+/-6.3 Area: 107552+/-164500
MPT32_pronase_Fract 9	866.4569++	APPPADPNAPPPPVIAPN	2	0.9996	1730.899171	866.456861	RT: 47.97+/-18.87 Area: 3674837682+/-5846015549
MPT32_pronase_Fract 9	796.3912++	TTGDPPFPGQPPPVAN	2	0.9984	1590.767822	796.391187	RT: 35+/-3.98 Area: 308608201+/-500464708
MPT32_pronase_Fract 9	879.4647++	APPPAPAPAPAEPAPAPAPA	2	0.9982	1756.914821	879.464686	RT: 32.44+/-0.59 Area: 14245392103+/-24539114849
MPT32_pronase_Fract 9	739.3697++	TTGDPPFPGQPPPVA	2	0.998	1476.724895	739.369723	RT: 33.77+/-7.11 Area: 259280409+/-444539704
MPT32_pronase_Fract 9	695.3435++	GDPPFPGQPPPVAN	2	0.9978	1388.672465	695.343509	RT: 34.89+/-6.1 Area: 5539088265+/-8629471564
MPT32_pronase_Fract 9	638.3220++	GDPPFPGQPPPVA	2	0.9973	1274.629538	638.322045	RT: 36.78+/-8.33 Area: 472843861+/-772449774
MPT32_pronase_Fract 9	577.9737+++	APPPADPNAPPPPVIAPN	3	0.9968	1730.899171	577.973666	RT: 47.95+/-18.97 Area: 1866456883+/-2946169666
MPT32_pronase_Fract 9	586.6455+++	APPPAPAPAPAEPAPAPAPA	3	0.9944	1756.914821	586.64555	RT: 32.57+/-0.45 Area: 6174432920+/-10641687371
MPT32_pronase_Fract 9	903.9547++	SKTTGDPPFPGQPPPVAN	2	0.9937	1805.894814	903.954683	RT: 33.57+/-5.56 Area: 22846997+/-14405011
MPT32_pronase_Fract 9	1044.0133++	AQPGDPNAAPPPADPNAPPPPV	2	0.9917	2086.011969	1044.01326	RT: 35.64+/-0.28 Area: 54589894+/-93480742
MPT32_pronase_Fract 9	609.3193++	PPFPGQPPPVAN	2	0.9915	1216.624058	609.319305	RT: 34.5+/-4.73 Area: 1692571500+/-2745484031
MPT32_pronase_Fract 9	619.6684+++	VAPPPAPAPAPAEPAPAPAPA	3	0.9862	1855.983235	619.668354	RT: 31.61+/-6.32 Area: 70629415+/-106074768
MPT32_pronase_Fract 9	809.4354++	APPPADPNAPPPPVIAP	2	0.9862	1616.856243	809.435398	RT: 39.49+/-1.39 Area: 173165888+/-276506081
MPT32_pronase_Fract 9	745.8673++	TGDPPFPGQPPPVAN	2	0.9859	1489.720144	745.867348	RT: 35.18+/-5.92 Area: 658886112+/-879683890
MPT32_pronase_Fract 9	602.9722+++	SKTTGDPPFPGQPPPVAN	3	0.9857	1805.894814	602.972214	RT: 33.67+/-5.45 Area: 9647879+/-9313381
MPT32_pronase_Fract 9	668.8484++	APPPADPNAPPPPV	2	0.9853	1335.682302	668.848427	RT: 31.46+/-0.52 Area: 7440955127+/-8318260587
MPT32_pronase_Fract 9	592.3271++	PNAPPPPVIAPN	2	0.9848	1182.639708	592.32713	RT: 32.88+/-0.61 Area: 121712224+/-182266215
MPT32_pronase_Fract 9	688.8459++	TGDPPFPGQPPPVA	2	0.982	1375.677216	688.845884	RT: 34.65+/-6.73 Area: 54657021+/-67935056
MPT32_pronase_Fract 9	803.3808++	GDPPFPGQPPPVANDT	2	0.9815	1604.747087	803.380819	RT: 38.78+/-4.97 Area: 379287309+/-296331013
MPT32_pronase_Fract 9	539.9594+++	APPPADPNAPPPPVIAP	3	0.9814	1616.856243	539.959357	RT: 39.54+/-1.4 Area: 54326258+/-87496050
MPT32_pronase_Fract 9	928.9989++	VAPPPAPAPAPAEPAPAPAPA	2	0.9798	1855.983235	928.998893	RT: 31.62+/-6.33 Area: 41490535+/-67333835
MPT32_pronase_Fract 9	696.3446+++	AQPGDPNAAPPPADPNAPPPPV	3	0.9773	2086.011969	696.344599	RT: 35.65+/-0.28 Area: 85089401+/-145989096
MPT32_pronase_Fract 9	860.4387++	KTTGDPPFPGQPPPVAN	2	0.9735	1718.862785	860.438669	RT: 31.58+/-4.91 Area: 23776785+/-32629110
MPT32_pronase_Fract 9	531.2632+++	TTGDPPFPGQPPPVAN	3	0.973	1590.767822	531.263217	RT: 35.24+/-3.85 Area: 3798368+/-4759463
MPT32_pronase_Fract 9	553.2693++	GDPPFPGQPPP	2	0.9699	1104.52401	553.269281	RT: 33.21+/-5.05 Area: 227793374+/-370828809
MPT32_pronase_Fract 9	463.8981+++	GDPPFPGQPPPVAN	3	0.9693	1388.672465	463.898098	RT: 35+/-6.23 Area: 11532231+/-18190265

MPT32_pronase_Fract 9	406.5486+++	PPFPGQPPPVAN	3	0.9603	1216.624058	406.548629	RT: 34.58+/-4.43 Area: 8739886+/-14571854
MPT32_pronase_Fract 9	446.2347+++	APPPADPNAPPPPV	3	0.9589	1335.682302	446.23471	RT: 31.48+/-0.46 Area: 885070487+/-992422562
MPT32_pronase_Fract 9	573.9615+++	KTTGDPPFPGQPPPVAN	3	0.9473	1718.862785	573.961538	RT: 31.58+/-5 Area: 9439565+/-10311970
MPT32_pronase_Fract 9	438.7323++	PGQPPPVAN	2	0.9317	875.450117	438.732334	RT: 16.72+/-0.07 Area: 9466569+/-12059698
MPT32_pronase_Fract 9	493.2489+++	TTGDPPFPGQPPPVA	3	0.8995	1476.724895	493.248908	RT: 33.65+/-7.01 Area: 205289+/-62379
MPT32_pronase_Fract 9	1044.2071+++	AQPGDPNAAPPPADPNAPPPPVIAPNAPQPVR	3	0.8535	3129.599582	1044.207137	RT: 72.49+/-34.7 Area: 733137+/-1223125
MPT32_pronase_Fract 9	587.9567+++	GDPPFPGQPPPVANDTR	3	0.7813	1760.848198	587.956675	RT: 30.51+/-10.46 Area: 10508742+/-11753091
MPT32_pronase_Fract 9	746.9116++	APPAPATPVAPPPPAAA	2	0.7337	1491.808565	746.911558	RT: 38.24+/-9.25 Area: 1122217+/-1096919
MPT32_pronase_Fract 9	881.4314++	GDPPFPGQPPPVANDTR	2	0.7132	1760.848198	881.431375	RT: 30.48+/-10.48 Area: 27434791+/-46926459
MPT32_pronase_Fract 9	425.8838+++	GDPPFPGQPPPVA	3	0.6952	1274.629538	425.883789	RT: 37.07+/-8.64 Area: 295920+/-184883
MPT32_pronase_Fract 9	497.5807+++	TGDPPFPGQPPPVAN	3	0.5111	1489.720144	497.580657	RT: 35.25+/-5.8 Area: 634243+/-769151
MPT32_pronase_Fract 9	459.5663+++	TGDPPFPGQPPPVA	3	0.4828	1375.677216	459.566348	RT: 34.57+/-6.3 Area: 107552+/-164500
MPT32_pronase_Fract 9	827.9380++	APPAPAT[+162.1]PVAPPPPAAA	2	0.4265	1653.861389	827.93797	RT: 31.46+/-0.21 Area: 10426544+/-17078471
MPT32_pronase_Fract 9	535.9230+++	GDPPFPGQPPPVANDT	3	0.4232	1604.747087	535.922972	RT: 38.84+/-4.74 Area: 349117+/-415574
MPT32_pronase_Fract 9	547.7725++	APDAGPPQRW	2	0.4124	1093.530492	547.772522	RT: 31.3+/-7.78 Area: 147872961+/-151495169

Supplemental Table 2. MS1 Signals of N-Terminal Glycopeptides from Pronase Digested MPT32

Peptide Sequence	Start,End Position	Observed Mass (Charge State)	MS1 Peak Area Fraction 7	idotp	MS1 Peak Area Fraction 8	idotp	MS1 Peak Area Fraction 9	idotp
DPEPAPPVPTT	1,11	560.7797++	6.65E+07	0.85	2.84E+06	0.67	3.01E+05	0.57
DPEPAPPVPT[+162.1]T	1,11	641.8061++	**1.50E+08**	**0.98**	2.59E+06	0.89	3.10E+04	0.91
DPEPAPPVPT[+162.1]T[+162.1]	1,11	722.8325++	**6.42E+06**	**0.95**	**2.62E+05**	**0.95**	1.64E+04	0.69
AASPPSTAAA	12,21	422.2140++	**6.69E+06**	**0.95**	**1.10E+06**	**0.96**	4.93E+04	0.53
AASPPST[+162.1]AAA	12,21	503.2404++	5.88E+05	0.29	4.40E+05	0.82	ND	0.00
AASPPS[+162.1]T[+162.1]AAA	12,21	584.2668++	1.21E+05	0.42	5.27E+04	0.83	**4.01E+04**	**0.98**
APPAPATPVAPPPPAAA	21,37	746.9116++	ND	0.00	1E+07	0.71	8E+05	0.73
APPAPAT[+162.1]PVAPPPPAAA	21,37	827.9380++	1E+06	0.52	**3E+07**	**0.98**	1E+03	0.43

Bold text indicates idotp scores at 0.95 or above (observed isotope distributions are >95% of expected value).

ND = Not Detected
